# Status Epilepticus Induces Vasogenic Edema via Tumor Necrosis Factor-α/ Endothelin-1-Mediated Two Different Pathways

**DOI:** 10.1371/journal.pone.0074458

**Published:** 2013-09-05

**Authors:** Ji-Eun Kim, Hea Jin Ryu, Tae-Cheon Kang

**Affiliations:** 1 Department of Anatomy and Neurobiology, College of Medicine, Hallym University, Chunchon, Kangwon-Do, Republic of Korea; 2 Institute of Epilepsy Research, College of Medicine, Hallym University, Chunchon, Kangwon-Do, Republic of Korea; Albany Medical College, United States of America

## Abstract

Status epilepticus (SE) induces vasogenic edema in the piriform cortex with disruptions of the blood-brain barrier (BBB). However, the mechanisms of vasogenic edema formation following SE are still unknown. Here we investigated the endothelin B (ET_B_) receptor-mediated pathway of SE-induced vasogenic edema. Following SE, the release of tumor necrosis factor-α (TNF-α) stimulated endothelin-1 (ET-1) release and expression in neurons and endothelial cells. In addition, TNF-α-induced ET-1 increased BBB permeability via ET_B_ receptor-mediated endothelial nitric oxide synthase (eNOS) activation in endothelial cells. ET_B_ receptor activation also increased intracellular reactive oxygen species by NADPH oxidase production in astrocytes. These findings suggest that SE results in BBB dysfunctions via endothelial-astroglial interactions through the TNF-α-ET-1-eNOS/NADPH oxidase pathway, and that these ET_B_ receptor-mediated interactions may be an effective therapeutic strategy for vasogenic edema in various neurological diseases.

## Introduction

Blood-brain barrier (BBB) disruption results in vasogenic edema posing a risk of hemorrhage in damaged vessels and contributing to a net increase in brain volume and pressure [1]. Loss of BBB integrity can result from an abrupt increase in intraluminal pressure and is influenced by the properties of cerebral tissues [2]. Status epilepticus (SE), defined as continuous seizure activity, is a medical emergency with significant mortality. SE results in neuronal damages, astroglial death, and BBB breakdown. Leakage of serum-derived components into the extracellular space is associated with hyperexcitability and seizure onset [3–9]. Thus, dysfunction of the BBB leads to epileptogenesis and contributes to the progression of epilepsy [3–5]. Despite the frequent occurrence of vasogenic edema and its undesirable consequences, the molecular mechanisms underlying vasogenic edema formation are still unknown.

The piriform cortex (PC) is one of the most vulnerable brain regions to seizure-induced vasogenic edema in the kainate, pilocarpine and other models of temporal lobe epilepsy [4,9,10]. We have previously shown in rats that the proinflammatory cytokine tumor necrosis factor (TNF)-α rapidly impairs endothelial cell functions via p65-Thr 485 nuclear factor-κB (NFκB) phosphorylation through TNFp75 receptor (TNFp75R) during SE-induced vasogenic edema formation [11]. Briefly, most activated microglia showed strong TNF-α immunoreactivity following SE. TNF p75 receptor expression was detected in endothelial cells as well as astrocytes. In addition, only p65-Thr435 phosphorylation was increased in endothelial cells accompanied by SMI-71 expression (an endothelial barrier antigen). Neutralization of TNF-α by soluble TNF p55 receptor (sTNFp55R) infusion attenuated SE-induced vasogenic edema and neuronal damages via inhibition of p65-Thr435 phosphorylation in endothelial cells [11]. Therefore, we have suggested that dysfunction of both endothelial cells and astrocytes may result in BBB breakdown and increase vascular permeability, leading to vasogenic edema. Based on our previous studies, it is likely that TNF-α/NFκB is one of potential signal pathways in SE-induced vasogenic edema formation. However, the signal downstreams of TNF-α/NFκB pathway in BBB disruption and vasogenic edema formation have not been fully clarified. Here, we demonstrate that TNF-α-mediated NFκB activation increased endothelial endothelin-1 (ET-1) expression and that endothelial ET-1 initiated SE-induced vasogenic edema formation through the dysfunction of astrocytes and endothelial cells via ET_B_ receptor-mediated NADPH oxidase and NOS activation, respectively. Therefore, we suggest that the TNF-α-NFκB-ET-1-ET_B_ receptor-NADPH oxidase (astrocyte)/NOS (endothelial cell) axes may play important roles in the neurovascular interactions during SE-induced vasogenic edema formations.

## Materials and Methods

### Experimental animals and chemicals

This study utilized male Sprague-Dawley (SD) rats (7 weeks old) obtained from Experimental Animal Center, Hallym University, Chunchon, Republic of Korea. The animals were provided with a commercial diet and water ad libitum under controlled temperature, humidity and lighting conditions (22 ± 2 °C, 55 ± 5% and a 12:12 light/dark cycle). Animal protocols were approved by the Institutional Animal Care and Use Committee of Hallym University (Chuncheon, Republic of Korea). The number of animals used and their suffering was minimized in all cases. All reagents were obtained from Sigma-Aldrich (St. Louis, MO, USA), except as noted.

### Surgery

For microdialysis, rats were anesthetized (Zolretil, 50 mg/kg I.M. Virbac Laboratories, France) and placed in a stereotaxic frame. Thereafter, a guide cannula was implanted in the PC (2 mm posterior; 5.5 mm lateral; −7.5 mm depth; flat skull position with the bregma as a reference), according to the rat brain atlas [12]. Rats were allowed 7 days to recover from the surgical procedure before the start of microdialysis. Animals were divided into five groups for intracerebroventricular drug infusion: (1) vehicle (n = 30), (2) sTNFp55R (2.5 μM, n = 30), (3) SN50 (a NFκB inhibitor; 20 μM, n = 30) (4) BQ-788 (an ET_B_ receptor antagonist; 3 pmol, n = 30), (5) Cav1 (an eNOS inhibitor; 5 μM, n = 30), and one group for intraperitoneal injection of apocynin (an NADPH oxidase inhibitor; 30 mg/kg, n = 30). The dosage of each compound did not affect seizure threshold, seizure score, mortality during SE, and BBB integrity in non-SE animals in the preliminary study. Animals were anesthetized (Zolretil, 50 mg/kg I.M. Virbac Laboratories, France) and placed in a stereotaxic frame. For the osmotic pump implantation, holes were drilled through the skull for introducing a brain infusion kit 1 (Alzet, USA) into the right lateral ventricle (1 mm posterior; 1.5 mm lateral; −3.5 mm depth), according to the atlas [12]. The infusion kit was sealed with dental cement and connected to an osmotic pump (1007D, Alzet, USA). The pump was placed in a subcutaneous pocket in the dorsal region. Animals received 0.5 µl/h of vehicle or compound for 1 week [13–15]. The compounds began to be immediately infused after surgery. Rats were allowed 3 days to recover from the surgical procedure before SE induction. Because the volume of vasogenic edema peaked at 2-3 days after SE in our previous studies [4,5], our experimental schedules inhibited the function of the related molecules from 3 days prior to SE to 4 days after SE when the volume of vasogenic edema peaked.

### SE induction

Three days after surgery, rats were treated with pilocarpine (380 mg/kg, I.P.) 20 min after atropine methylbromide (5 mg/kg, I.P.) and were placed in individual observation chambers where seizure activity was scored according to the system of Racine [16]. Approximately 90% of pilocarpine-treated animals entered SE within 20 to 30 min of the administration of pilocarpine and exhibited continuous seizure activity between 2 and 5 on the Racine scale (including akinesia, facial automatisms, limbic seizures consisting of forelimb clonus with rearing, salivation, masticatory jaw movements, and falling). One – two animals in each group died during SE. Diazepam (10 mg/kg, I.P.) was administered 2 hr after onset of SE and repeated, as needed. Age-matched animals were used as non-SE experienced controls (non-SE animals, n = 30). Non-SE animals received saline in place of pilocarpine [17].

### Microdialysis

One day before SE induction, a microdialysis probe (CMA 12; cut-off, 100,000 Da, membrane diameter, 0.5 mm, membrane length, 2 mm) was inserted via the microdialysis guide cannula into the PC of freely moving rats (n = 7). The probe was perfused with Ringer’s solution (in mM: NaCl 147, CaCl2 1.26, KCl 2.5, and MgCl2 1.18 in sterile water, pH 7.4), at a constant flow rate of 1 µl/min via a microperfusion pump (CMA/100 microinjection pump, Carnegie Medicine, Sweden) for 4 hr before/after SE induction. Microdialysis samples were collected 240 μl before and after SE induction, respectively. Samples were transferred to -80 °C freezer and stored until analysis. At the end of the experimental period, the animals were killed, and their brains were fixed to identify the location of the microdialysis probe in the PC. Only the animals with the probe tip in the designated location were included in the analysis.

### Enzyme-linked immunosorbent assay (ELISA) and nitric oxide (NO) assay

The concentration of TNF-α, and NO in perfusates were measured using the Quantikine^®^ ELISA kits (R&D Systems, Abingdon, UK), and a nitrate/nitrite fluorometric assay kit (Cayman chemical company, USA), according to the manufacturer’s instructions. The concentration of big ET-1 in perfusates was measured using the rat big ET-1 ELISA kit (Enzo Life Science), according to the manufacturer’s instructions. Big ET-1 (39 amino acid sequences) is processed to ET-1 (21 amino acid sequences). Big ET-1 is more stable than ET-1. However, all ET-1 assay kits also detect ET-2 and ET-3. Therefore, the concentration of big ET-1 in perfusates was measured using the rat big ET-1 ELISA kit.

### Tissue processing

At designated time points, animals were perfused transcardially with phosphate-buffered saline (PBS) followed by 4% paraformaldehyde in 0.1 M phosphate buffer (PB, pH 7.4) under urethane anesthesia (1.5 g/kg, I.P.). The brains were removed and postfixed in the same fixative for 4 hr. The brains were infiltrated with 30% sucrose overnight, frozen and sectioned with a cryostat at 30 μm and consecutive sections were contained in six-well plates containing PBS. Every sixth section in the series throughout the entire PC was used for stereological study [4]. For western blot, tissues were homogenized in 50 mM Tris containing 50 mM HEPES (pH 7.4), ethylene glycol tetraacetic acid (EGTA, pH 8.0), 0.2% Tergitol type NP-40, 10 mM ethylenediaminetetraacetic acid (EDTA, pH 8.0), 15 mM sodium pyrophosphate, 100 mM β-glycerophosphate, 50 mM NaF, 150 mM NaCl, 2 mM sodium orthovanadate, 1 mM phenylmethylsulfonyl fluoride (PMSF), and 1 mM dithiothreitol (DTT). After centrifugation, the protein concentration in the supernatant was determined using a Micro BCA Protein Assay Kit with bovine serum albumin as the standard (Pierce Chemical, Rockford, IL, USA).

### Vasogenic edema measurement

To confirm vasogenic edema, the free-floating sections were incubated with horse anti-rat IgG (Vector, USA). After washing three times for 10 min with PBS, sections were incubated in ABC complex (Vector, USA, diluted 1:200). The sections were visualized with 3,3′-diaminobenzidine (DAB) in 0.1 M Tris buffer and mounted on the gelatin-coated slides. To measure vasogenic edema, the volume of the anti-rat IgG positive region in the PC was estimated according to a formula based on the modified Cavalieri method: V = Σa × t_nom_ × 1/ssf, where a is area of the region of the delineated subfield measured by AxioVision Rel. 4.8 software, t_nom_ is the nominal section thickness (30 μm in this study), and ssf is the fraction of the sections sampled or section sampling fraction (1/6 in this study). The subfield areas were delineated with a 2.5 × objective lens [4,17]. The volumes are reported in mm^3^.

### Double immunofluorescence study

Table 1 provides a list of the primary antibodies and lectins used. Sections were incubated in a mixture of antisera (or lectin) in PBS containing 0.3% Triton X-100 overnight at room temperature. After washing three times for 10 min with PBS, the sections were also incubated in a mixture of FITC- and Cy3-conjugated secondary antisera (or streptavidin, 1:250, Amersham, USA) for 2 hr at room temperature. The sections were washed three times for 10 min with PBS, and mounted on gelatin-coated slides. All images were captured using an AxioImage M2 microscope and AxioVision Rel. 4.8 software. Fluorescence intensity was measured using computer-assisted image analysis program (The University of Texas ImageTool program V. 3.0 and AxioVision Rel. 4.8 software) [18].

**Table 1:** Primary antibodies used in the present study.

Antigen/lectin	Host	Manufacturer (catalog number)	Dilution used
Aquaporin 4 (AQP4)	Rabbit	Almone labs (AQP-004)	1:500 (IF) 1:5000 (WB)
Dystrophin	Rabbit	Abcam (ab15277)	1:500 (IF) 1:5000 (WB)
Endothelial nitric oxide synthase (eNOS)	Rabbit	Abcam (ab66127)	1:500 (IF) 1:1000 (WB)
Endothelin-1 (ET-1)	Rabbit	Abbiotec (250633)	1:500 (IF)
Endothelin B receptor (ET_B_ receptor)	Rabbit	Millipore (AB3284)	1:200 (IF) 1:1000 (WB)
Glial fibrillary acidic protein (GFAP)	Mouse	Chemicon (MAB3402)	1:5000 (IF)
4-hydroxynonenal (4-HNE)	Rabbit	Alpha diagnostic (HNE11-2)	1:200 (IF)
Neuronal nuclear antigen (NeuN)	Mouse	Chemicon (MAB377)	1:1000 (IF)
Phospho-p65-Thr435 NF-κB	Rabbit	Abcam (ab31472)	1:200 (IF) 1:200 (WB)
Nitrotyrosine (NT)	Rabbit	Millipore (AB5411)	1:500 (IF)
p47phox	Rabbit	Abbiotec (252159)	1:200 (IF) 1:1000 (WB)
Ricinus Communis Agglutinin I (RCA I)	-	Vector (B-1085)	1:250 (IF)
SMI-71	Mouse	Covunce (SMI-71R)	1:5000 (IF)
Tumor necrosis factor p75 receptor (TNFp75 receptor)	Rabbit	Abcam (ab15563)	1:200 (IF) 1:500 (WB)

IF, Immunofluorescence; WB, Western blot.

### Western blot

Aliquots containing 20 μg total protein were boiled in a loading buffer containing 150 mM Tris (pH 6.8), 300 mM DTT, 6% sodium dodecyl sulfate (SDS), 0.3% bromophenol blue, and 30% glycerol. Each aliquot was loaded into a 10% polyacrylamide gel. After electrophoresis, gels were transferred to nitrocellulose transfer membranes (Schleicher and Schuell BioScience Inc.). To reduce background staining, the filters were incubated with 5% nonfat dry milk in TBS containing 0.1% Tween 20 for 45 min, followed by incubation first with the primary antibody (Table 1) and subsequently with an HRP-conjugated secondary antibody. Western blotting was performed with an ECL Western Blotting Detection Kit (Amersham) [19]. Intensity measurements were represented as the mean gray-scale value on a 256 gray-level scale [19].

### RNA extraction, reverse transcription and quantitative real-time PCR

Brain tissues were homogenized and total RNA was extracted using Trizol Reagents, according to the manufacturer’s protocol (Ambion, TX, USA). One μg of total RNA was reverse transcribed into first-strand cDNA using a PrimerScript 1^st^ strand cDNA synthesis kit (Takara, Shiga, Japan). Quantification of mRNA expression was performed in triplicate using a SYBR Green SuperMix (Bioneer, Taejon, South Korea) in a two-step PCR reaction procedure, performed with the MyiQ Single Color Real-Time PCR Detection System (Bioneer, Taejon, South Korea). Ten microliters cDNA from the RT-reaction was used as the template for the quantitative real-time PCR reaction with a final PCR reaction volume of 50 μl. The 5′ and 3′ gene-specific PCR primer concentrations were 10 pM each. Real-time PCR primers were designed using Primer3 software (Whitehead Institute, MA, USA) according to the coding sequences of each target gene (Table 2). To discriminate DNA contamination from cDNA, initial PCR reaction with GAPDH primer (designed by spanning intron) was confirmed using by direct visualization from electrophoresis. Other primers were selected after the specificity was confirmed by primer-BLAST software (NIH, MD, USA) and the single PCR band was directly visualized by agarose gel to avoid primer dimer formation in the PCR reactions. After initial denaturation at 95 °C for 3 min, 40 cycles of primer annealing and elongation were performed at 60 °C for 45 s, followed by denaturation at 95 °C for 10 s. Fluorescence emission data were captured, and mRNA levels were quantified using the threshold cycle value (Ct). To compensate for variations in input RNA amounts and efficiency of reverse transcription, qPCR data for mRNA for each sample were normalized to the house keeping protein GAPDH determined from the same experiment.

**Table 2:** Coding sequences of each target gene used in the present study.

Target gene	Sequences
AQP4	Forward CATTTGTTTGCAATCAATTATAC
	Reverse GACAGAAGACATACTCGTAAAGT
Dystrophin	Forward TGGGCAGAGCGATGGAGTCCT
	Reverse ACCATGCGGGGGTCAGGAGTT
eNOS	Forward TATTTGATGCTCGGGACTGC
	Reverse AAGATTGCCTCGGTTTGTTG
ET-1	Forward GACCAGCGTCCTTGTTCCAA
	Reverse TTGCTACCAGCGGATGCAA
ET_B_ receptor	Forward GATACGACAACTTCCGCTCCA
	Reverse GTCCACGATGAGGACAATGAG
GAPDH	Forward TGGAGTCTACTGGCGTCTT
	Reverse TGTCATATTTCTCGTGGTTCA
p47phox	Forward TCACCGAGATCTACGAGTTC
	Reverse TCCCATGAGGCTGTTGAAGT

### Data analysis

All data obtained from the quantitative measurements were analyzed using Student’s t-test or one-way ANOVA to determine statistical significance. Turkey’s test was used for post-hoc comparisons. A p-value below 0.05 was considered statistically significant [4].

## Results

### TNF-α-TNFp75R-NFκB-mediated BBB dysfunction

**Figure 1:**
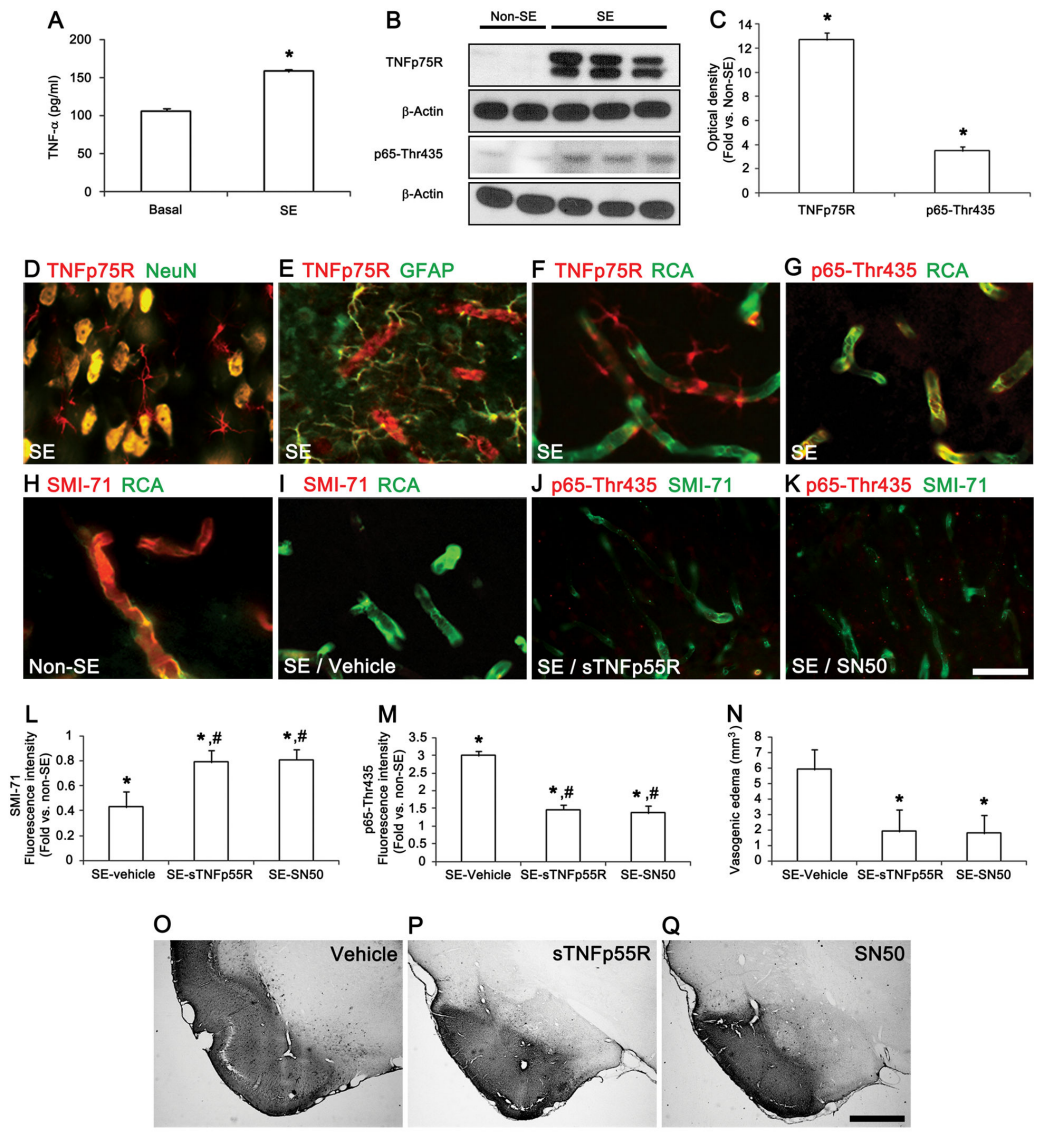
The roles of TNF-α in SE-induced vasogenic edema in the PC. (**A**) The extracellular TNF-α concentration after SE (mean ± s.d., n = 5): *P < 0.05 versus the basal level; paired Student’s t-test. (**B** and **C**) Quantification of western blots for TNF-α protein expression and p65-Thr435 NFκB phosphorylation 12 h after SE (means ± s.e.m., n = 5, respectively); *P < 0.05 by Student’s t-test. (**D**–**I**) Immunofluorescence data for TNFp75R, p65-Thr435 NFκB phosphorylation and SMI-71 12 h after SE. (**J**–**K**) Effects of TNFp55R and SN50 on p65-Thr435 NF-κB phosphorylation and SMI-71 immunoreactivity. (**L**–**M**) Quantification of the fluorescence intensities of SMI-71 expression and p65-Thr435 NFκB phosphorylation 12 h after SE (means ± s.e.m., n = 5, respectively); *P < 0.05 versus non-SE animals; #P < 0.05 versus vehicle-treated animals; one-way analysis of variance (ANOVA) followed by Tukey’s test. (**N**–**Q**) Quantification of vasogenic edema attenuation by sTNFp55R and SN50 3 days after SE (means ± s.e.m., n = 5, respectively); *P < 0.05 versus vehicle treated animals by one-way ANOVA followed by Tukey’s test. Scale bars: **D–K**, 25 μm; **O-Q**, 400 μm.

We first investigated whether SE affects TNF-α release. We implanted microdialysis systems in freely moving rats before and after SE and measured the extracellular TNF-α concentration. The basal concentration level of TNF-α was 106.1 ± 3.5 pg/ml in the PC. After SE, the TNF-α concentration rose to 158.1 ± 2.9 pg/ml (Figure 1A). Consistent with our previous study [11], TNFp75R expression and p65-Thr435 NFκB phosphorylation were rarely detected in the PC of non-SE animals (data not shown). Twelve h after SE, TNFp75R protein expression and p65-Thr435 NFκB phosphorylation were also significantly increased in the PC (Figure 1B and C). Immunohistochemical studies revealed that that TNFp75R immunoreactivity was up-regulated in neurons, astrocytes and endothelial cells (Figure 1D–F), and p65-Thr435 NFκB phosphorylation increased in endothelial cells (Figure 1G). In contrast, SMI-71 (an endothelial barrier antigen) immunoreactivity decreased to 0.43-fold that of non-SE animals (Figure 1H, I and L). To confirm the effect of TNF-α- NFκB mediated signaling on BBB breakdown, we neutralized TNF-α by sTNFp55R infusion or inhibited the NFκB activity by SN50 prior to SE. sTNFp55R infusion and SN50 pretreatment effectively inhibited p65-Thr435 NF-κB phosphorylation and the loss of SMI-71 immunoreactivity in endothelial cells induced by SE, compared to the vehicle (Figure 1J, K and M). The volume of vasogenic edema in the PC of vehicle-pretreated animals was 5.9 mm^3^, and sTNFp55R and SN50 pretreatment attenuated the volume of vasogenic edema to 1.9 mm^3^ and 1.78 mm^3^, respectively (Figure 1N–Q). These findings indicate that TNF-α-mediated signals may play an important role in SE-induced vasogenic edema formation via p65-Thr435 NF-κB phosphorylation.

### TNF-α/NFκB-mediated regulation in ET-1 systems following SE

**Figure 2:**
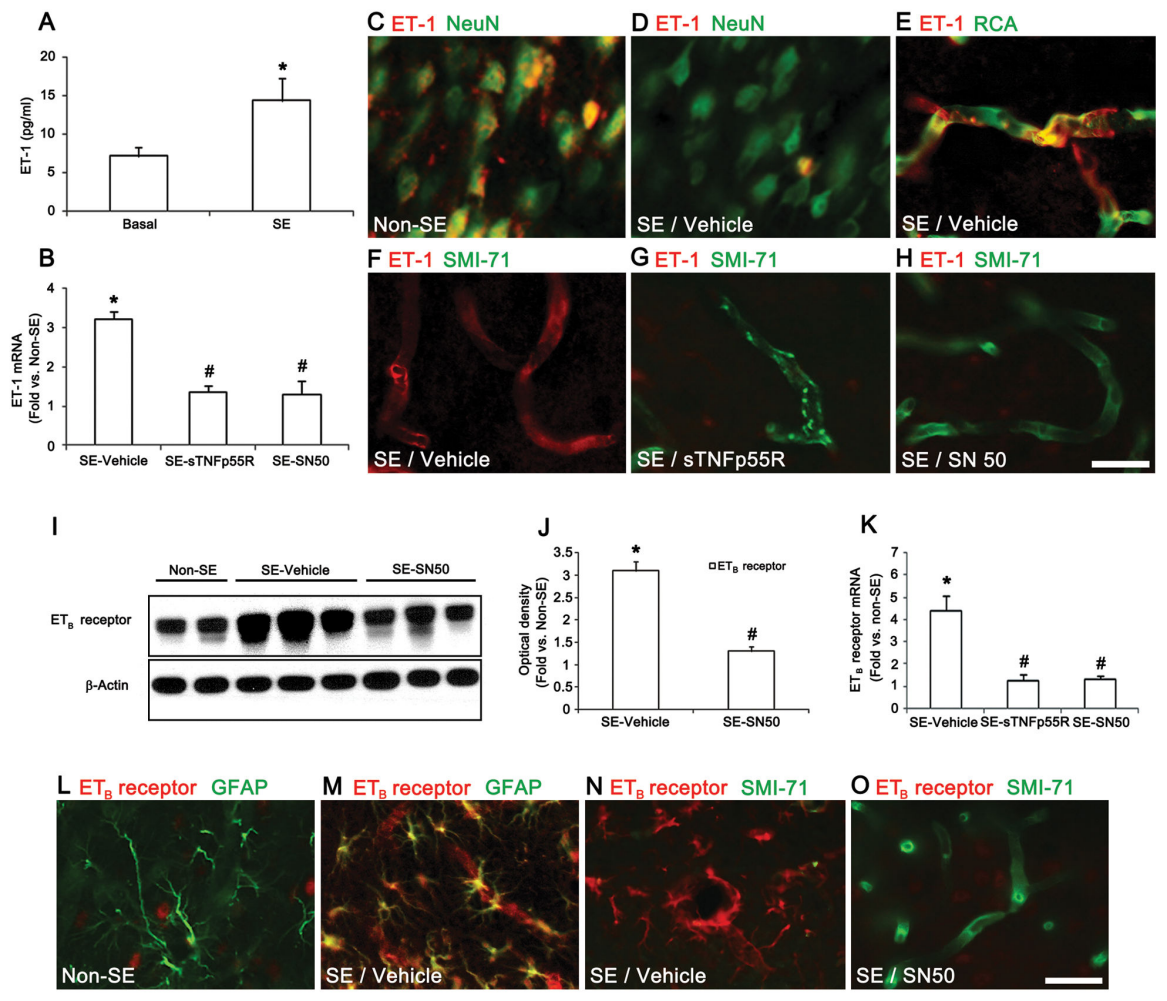
TNF-α/NFκB-mediated ET-1 release and expression in the PC following SE. (**A**) The extracellular ET-1 concentration in the PC after SE (mean ± s.d., n = 5): *P < 0.05 versus basal level; paired Student’s t-test. (**B**) The effect of sTNFp55R, and SN50 pretreatment on ET-1 mRNA expression 12 h after SE (means ± s.e.m., n = 5, respectively); *P < 0.05 versus non-SE animals; #P < 0.05 versus vehicle-treated animals; one-way ANOVA followed by Tukey’s test. (**C**–**F**) ET-1 expression in neurons and endothelial cells 12 h after SE. (**G**–**H**) Effects of sTNFp55R and SN50 pretreatment on ET-1 expression and SMI-71 immunoreactivity 12 h after SE. (**I**–**K**) Quantification of ET_B_ receptor levels by western blotting and qRT-PCR in the PC 12 h after SE (means ± s.e.m., n = 5, respectively); *P < 0.05 versus non-SE animals; #P < 0.05 versus vehicle-treated animals; one-way ANOVA followed by Tukey’s test. (**L**–**O**) Effect of SN50 on ETB receptor expression and SMI-71 immunoreactivity 12h after SE. Scale bars: **C–H**, **L-O**, 25 μm.

TNF-α stimulates ET-1 release and ET-1 expression in rat brain capillary [20,21]. Therefore, we investigated whether TNF-α/NFκB-mediated signals result in ET-1 expression/release induced by SE. The basal level of big ET-1 concentration was 7.1 ± 1.1 pg/ml in the PC. SE elevated big ET-1 to 14.3 ± 2.9 pg/ml (Figure 2A), and increased ET-1 mRNA by 3.92-fold over that of the non-SE animals (Figure 2B). Immunohistochemistry revealed an up-regulation of endothelial ET-1 expression (Figure 2C–F). Pretreatment by both sTNFp55R and SN50 significantly inhibited the up-regulation of ET-1 mRNA expression compared to the vehicle infusion (Figure 2B). Furthermore, both sTNFp55R and SN50 reduced endothelial ET-1 protein expression, and preserved SMI-71 immunoreactivity, compared to vehicle infusion (Figure 2G and H). Taken together, our findings indicate that following SE, TNF-α/TNFp75R/NFκB signals may induce ET-1 expression in endothelial cells. To determine the role of ET-1 in SE-induced vasogenic edema, we investigated whether SE affects levels of the ETB receptor, as the ET_B_ receptor is predominantly expressed in brain parenchyma [22,23]. SE increased ETB receptor protein and mRNA expression by 3.1- and 4.5-fold, respectively, over that of the non-SE animals (Figure 2I–K). In the non-SE animals, ETB receptor immunoreactivity was weakly observed in a few PC neurons (Figure 2L). Twelve hr after SE, ETB receptor expression increased in astrocytes and endothelial cells (Figure 2M). In addition, ETB receptor expressing endothelial cells showed a reduction in SMI-71 immunoreactivity (Figure 2N). SN50 pretreatment preserved SMI-71 immunoreactivity in endothelial cells, and reduced ET_B_ receptor expression in endothelial cells and astrocytes induced by SE compared to vehicle (Figure 2O). Thus, our data suggest that, following SE, the TNF-α-NF-κB signaling pathway may be upstream of ET-1 and ETB receptor inductions in astrocytes and endothelial cells.

### ET_B_ receptor-mediated endothelial dysfunction via NOS activation

**Figure 3:**
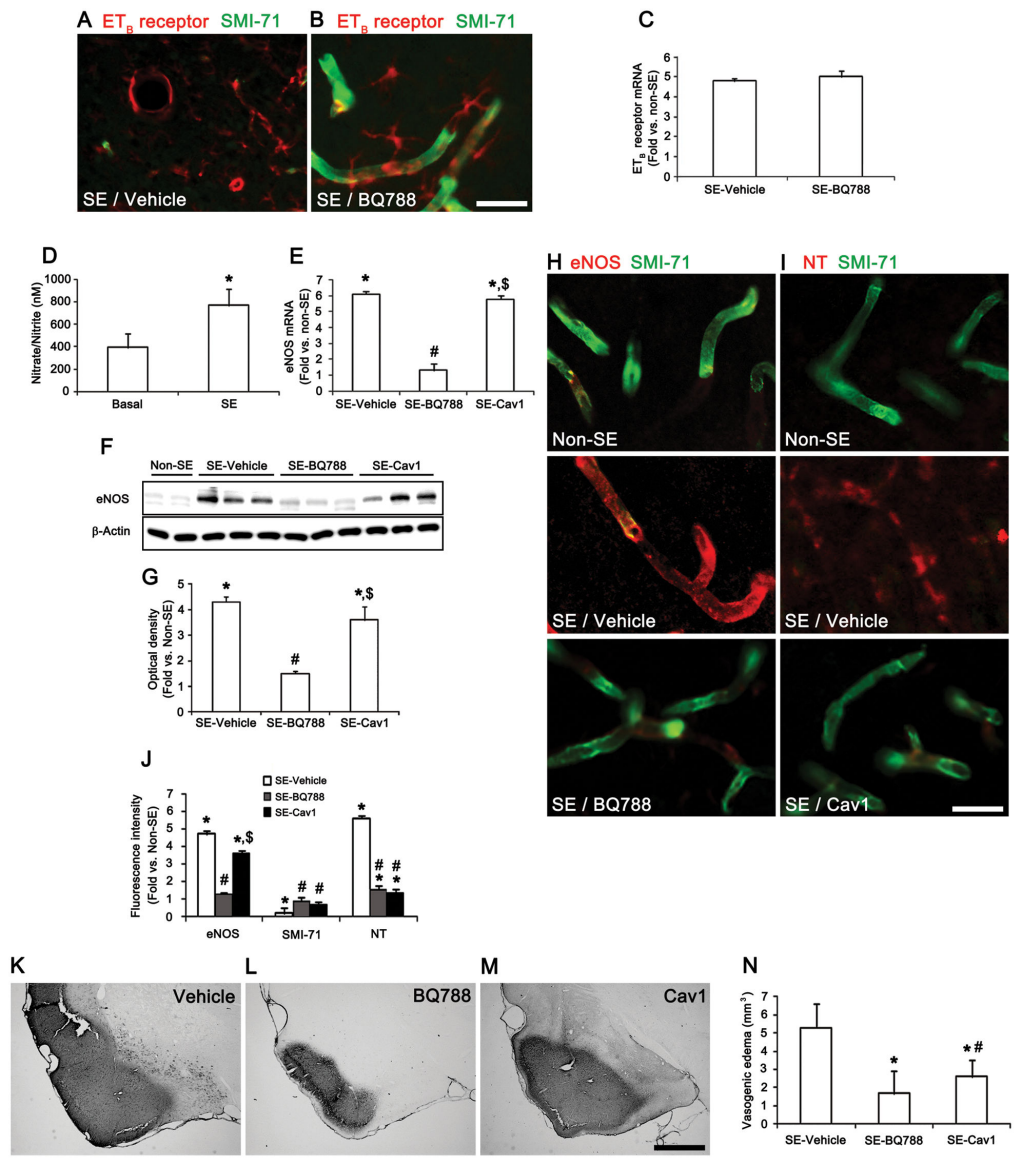
SE-induced vasogenic edema formation via the ETB receptor-mediated eNOS pathway. (**A**–**B**) Effect of BQ788 on SMI-71 expression and ETB receptor expression 12 h after SE. (**C**) Effect of BQ788 on ET_B_ receptor mRNA expression 12 h after SE (means ± s.e.m., n = 5, respectively); paired Student’s t-test. (**D**) Nitrate/nitrite (NO products) concentration in the PC after SE (mean ± s.d., n = 5): *P < 0.05 versus basal level; paired Student’s t-test. (**E**–**G**) Effects of BQ788 and Cav1-peptide on eNOS mRNA/protein expression 12 h after SE (means ± s.e.m., n = 5, respectively); *P < 0.05 versus non-SE animals; #P < 0.05 versus vehicle-treated animals; $P < 0.05 versus BQ788-treated animals; one-way ANOVA followed by Tukey’s test. (**H**–**J**) Effects of of BQ788 and Cav1-peptide on eNOS, SMI-71 and NT expression 12 h after SE (means ± s.e.m., n = 5, respectively); *P < 0.05 versus non-SE animals; #P < 0.05 versus vehicle-treated animals; $P < 0.05 versus BQ788-treated animals; one-way ANOVA followed by Tukey’s test. (**K**–**N**) Quantification of vasogenic edema formation 3days after SE (means ± s.e.m., n = 5, respectively); *P < 0.05 versus vehicle-treated animals; #P < 0.05 versus BQ788-treated animals; one-way ANOVA followed by Tukey’s test. Scale bars: **A, B, H** and **I**, 25 μm; **K-M**, 400 μm.

To investigate whether SE induces vasogenic edema formation via ET_B_ receptor-mediated pathways, BQ788 (an ET_B_ receptor antagonist) was applied before SE induction. BQ788 pretreatment significantly attenuated reduction in SMI-71 immunoreactivity induced by SE, but did not affect ET_B_ receptor expression in endothelial cells or astrocytes (Figure 3A–C). ET-1 triggers a signaling cascade that leads to the production of NO derived from endothelial nitric oxide synthase (eNOS), inducible NOS (iNOS), and neuronal NOS (nNOS) in endothelial cells, astrocytes/microglia, and neurons, respectively. This activation of NOS increases NO synthesis, which affects BBB permeability in various pathophysiological conditions [24,25]. NO activates matrix metalloproteinases [26] that hydrolyze tight junction proteins in the brain endothelial cells [27]. The present data showed that SE increased total nitrate/nitrite (NO products) levels from 394.9 ± 117.8 nM to 768.5 ± 141.0 nM (Figure 3D). qRT-PCR data also revealed that only eNOS mRNA and its expressed protein increased 5.9- and 4.1-fold, respectively, over that of non-SE animals 12 h after SE (Figure 3E–G). BQ788 pretreatment effectively prevented the SE-induced upregulation of eNOS protein/mRNA expression level (Figure 3E–G). However, the Cav1-peptide (an eNOS inhibitor) did not inhibit this up-regulation of eNOS protein and its mRNA expression levels (Figure 3E–G). Immunohistochemistry revealed that SE significantly elevated eNOS protein expression in endothelial cells. eNOS-positive endothelial cells exhibited an absence of SMI-71 immunoreactivity, but strong nitrotyrosine (NT, a marker for NO-dependent reactive nitrogen species-mediated damage) immunoreactivity (Figure 3H–J). BQ788 pretreatment effectively prevented the SE-induced up-regulations of eNOS protein expression and NT immunoreactivity and reduction in SMI-71 immunoreactivity in the PC (Figure 3H–J). Cav1-peptide inhibited the up-regulation of NT immunoreactivity and reduction in SMI-71 immunoreactivity, whereas it did not affect eNOS protein expression induced by SE (Figure 3H–J). Furthermore, BQ788 and Cav1-peptide pretreatment reduced SE-induced vasogenic edema to 1.63 and 2.6 mm^3^, respectively (Figure 3K–N). However, Cav1-peptide pretreatment was less effective in inhibiting SE-induced vasogenic edema formation than BQ788 pretreatment (Figure 3N). Thus, our findings indicate that ET_B_ receptor-mediated NOS activation may induce SE-induced vasogenic edema.

### ET_B_ receptor-mediated down-regulation of dystrophin and AQP4 expression in astrocytes independent of NOS activity

**Figure 4:**
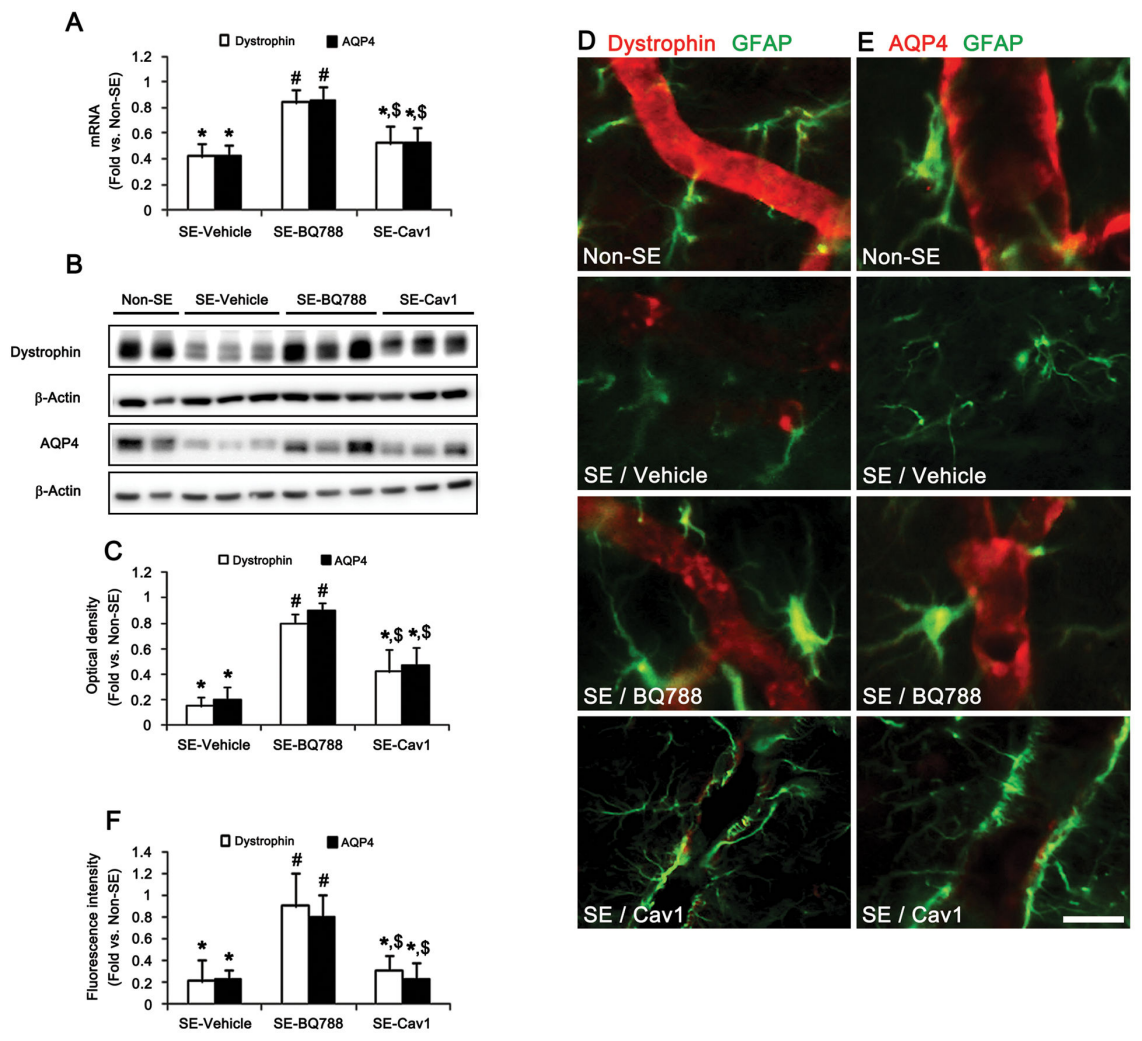
ETB receptor-mediated reduction of dystrophin and AQP4 expression in astrocytes. (**A**–**C**) Effects of BQ788 and Cav1-peptide on dystrophin and AQP4 mRNA/protein expression levels 12 h after SE (means ± s.e.m., n = 5, respectively); *P < 0.05 versus non-SE animals; #P < 0.05 versus vehicle-treated animals; $P < 0.05 versus BQ788-treated animals; one-way ANOVA followed by Tukey’s test. (**D**–**F**) Effects of BQ788 and Cav-1 peptide on dystrophin and AQP4 expression in astrocytes 12 h after SE (means ± s.e.m., n = 5, respectively); *P < 0.05 versus non-SE animals; #P < 0.05 versus vehicle-treated animals; $P < 0.05 versus BQ788-treated animals; one-way ANOVA followed by Tukey’s test. Scale bar: **D** and **E**, 12.5 μm.

Dystrophin plays a role in establishing endothelial polarity and as the anchor protein for AQP4. Thus dystrophin deficiency leads to severe BBB breakdown accompanied by impaired AQP4 expression in perivascular astroglial end-feet [28,29]. In our previous studies [4,5], dystrophin immunoreactivity has been significantly reduced in the PC 12 hr after SE when vasogenic edema and the down-regulation of SMI-71/AQP4 immunoreactivity have been observed. Therefore, it is likely that the dysfunction of dystrophin by ET-1 may result in BBB breakdown and increase vascular permeability, leading to vasogenic edema. We investigated whether the ET-1 system affects vascular permeability via dysfunction of the dystrophin/AQP4 complex induced by SE. Following SE, dystrophin and AQP4 mRNA expression levels decreased by 0.42- and 0.43-fold, respectively, compared to that of the of non-SE animals (Figure 4A). Dystrophin and AQP4 protein expression levels were also reduced by 0.17- and 0.19-fold of the non-SE animal’s levels (Figure 4B and C). BQ788 pretreatment effectively prevented the reduction in dystrophin/AQP4 mRNA/protein expression levels induced by SE (Figure 4A–C). However, Cav1-peptide pretreatment did not attenuate SE-induced reductions in dystrophin/AQP4 expression following SE (Figure 4A–C). Immunohistochemistry showed that SE reduced dystrophin/AQP4 immunoreactivity within the processes and cell bodies of astrocytes and endothelial cells (Figure 4D–F). BQ788, but not Cav-1 peptide, pretreatment effectively prevented the SE-induced reductions in dystrophin/AQP4 immunoreactivity (Figure 4F). These findings indicate that ET_B_ receptor activation may result in the dysfunction of the dystrophin/AQP4 complex in astrocytes through an NO-independent pathway.

### ET_B_ receptor-mediated astroglial function via NADPH oxidase activation

**Figure 5:**
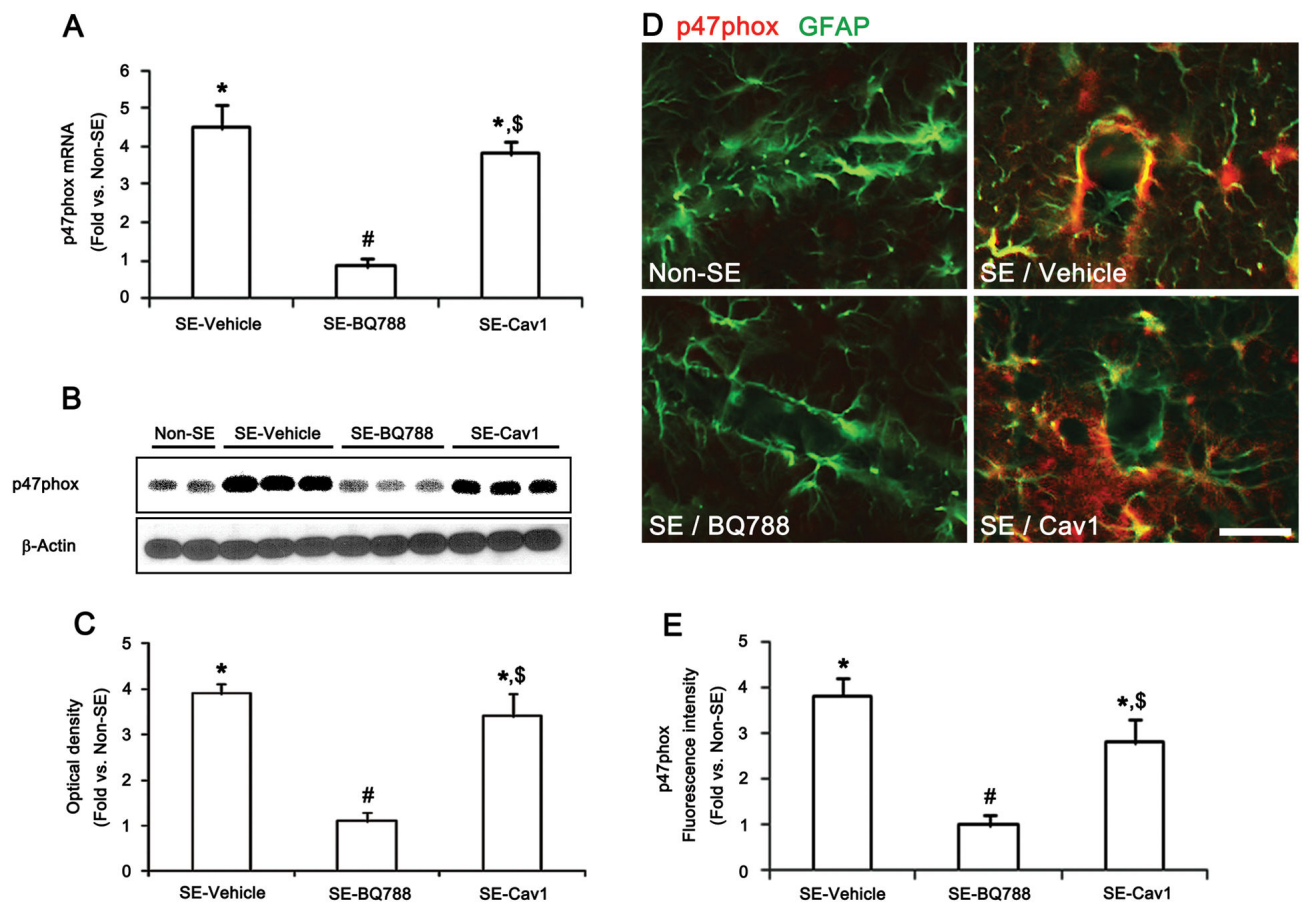
ETB receptor-mediated p47phox expression in astrocytes. (**A**–**C**) Effects of BQ788 and Cav1-peptide on p47phox mRNA/protein expression level 12 h after SE (means ± s.e.m., n = 5, respectively); *P < 0.05 versus non-SE animals; #P < 0.05 versus vehicle-treated animals; $P < 0.05 versus BQ788-treated animals; one-way ANOVA followed by Tukey’s test. (**D**–**E**) Effects of BQ788 and Cav-1 peptide on p47phox expression in astrocytes 12 h after SE (means ± s.e.m., n = 5, respectively); *P < 0.05 versus non-SE animals; #P < 0.05 versus vehicle-treated animals; $P < 0.05 versus BQ788-treated animals; one-way ANOVA followed by Tukey’s test (**E**). Scale bar: **D**, 25 μm.

**Figure 6:**
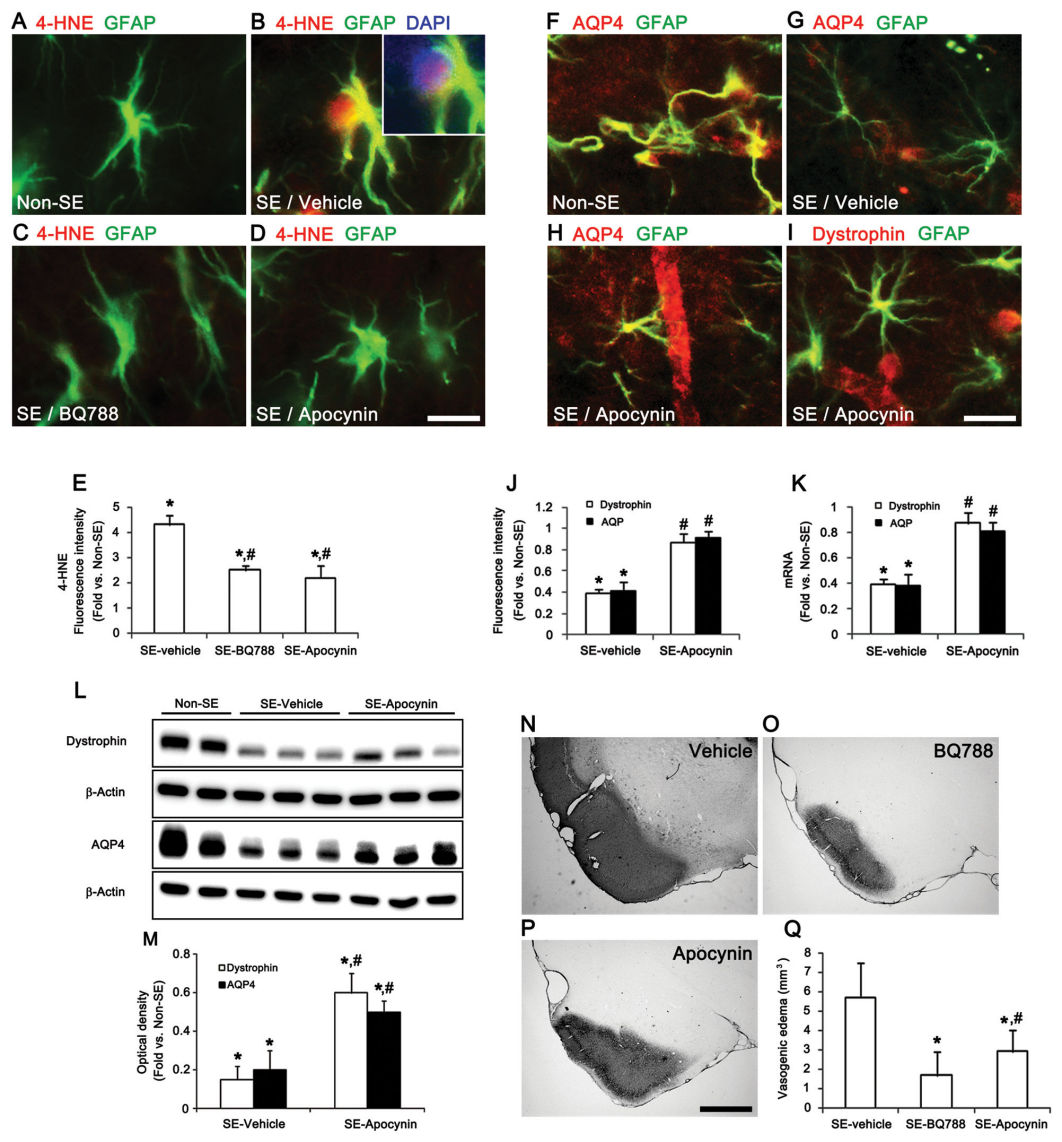
SE-induced vasogenic edema formation via ETB receptor-mediated NADPH oxidase pathway. (**A**–**E**) Effects of BQ788 and apocynin on SE-induced up-regulation of 4-HNE immunoreactivity in astrocytes 12 h after SE (means ± s.e.m., n = 5, respectively); *P < 0.05 versus non-SE induced animals; #P < 0.05 versus vehicle-treated animals; one-way ANOVA followed by Tukey’s test. (**F**–**J**) Effects of BQ788 and apocynin on dystrophin and AQP4 expression 12h after SE (means ± s.e.m., n = 5, respectively); *P < 0.05 versus non-SE induced animals; #P < 0.05 versus vehicle-treated animals; one-way ANOVA followed by Tukey’s test. (**K**–**M**) Effect of apocynin on dystrophin and AQP4 mRNA/protein expression levels after SE (means ± s.e.m., n = 5, respectively); *P < 0.05 versus non-SE animals; #P < 0.05 versus vehicle-treated animals; one-way ANOVA followed by Tukey’s test. (**N**–**Q**) Quantification of the attenuation of vasogenic edema formation by BQ788 and apocynin in the PC (means ± s.e.m., n = 5, respectively); *P < 0.05 versus vehicle treated animals; #P < 0.05 versus BQ788-treated animals; one-way ANOVA followed by Tukey’s test. Scale bars: **A-D**, 12.5 μm; **insertion** in **B**, 10 μm; **F–I**, 25 μm; **N–P**, 400 μm.

The NADPH oxidase enzyme system is the major source of reactive oxygen species (ROS) in various cells [30–32]. NADPH oxidase is a multi-component enzyme and is composed of three cytosolic proteins, p40phox, p47phox, and p67phox, and at least two membrane proteins, including gp91phox and p22phox [33]. The assembly of the NADPH complex is regulated by p47phox [34]. Because ET-1 activates NADPH oxidase [35,36], it is likely that ET_B_ receptor-mediated NADPH oxidase activation will prompt astroglial dysfunction, thereby inducing vasogenic edema following SE. To test this hypothesis, we investigated the change in p47phox expression levels following SE. SE increased p47phox mRNA/protein expression levels by 4.51- and 3.93-fold, respectively, over those of non-SE animals’ levels (Figure 5A–C). Immunohistochemistry revealed that SE up-regulated p47phox protein expression in astrocytes (Figure 5D, p < 0.05 vs. non-SE animals). BQ788 pretreatment effectively prevented the up-regulation of astroglial p47phox mRNA/protein expression induced by SE (Figure 5A–E). However, Cav1-peptide pretreatment did not affect SE-induced astroglial p47phox expression (Figure 5A–E). Astrocytes positive for p47phox also showed 4-hydroxynonenal (4-HNE, a marker for a ROS-dependent lipid peroxidation) immunoreactivity following SE (Figure 6A and B). BQ788 and apocynin (an NADPH oxidase inhibitor) pretreatment effectively prevented the up-regulation of 4-HNE immunoreactivity (Figure 6C–E). Apocynin pretreatment also preserved dystrophin/AQP4 expression in astrocytes and endothelial cells following SE (Figure 6F–M) and attenuated SE-induced vasogenic edema to 2.9 mm^3^ (Figure 6N–Q). These findings indicate that ET_B_ receptor activation in astrocytes may generate ROS by NADPH oxidase, initiating vasogenic edema formation via the dysfunction of the dystrophin/AQP4 complex.

## Discussion

The novel finding in the present study is that the TNFα-NFκB-ET-1-ET_B_ receptor axis showed cell specific responses to NOS (endothelial cell)/ NADPH oxidase (astrocyte) activation in the PC following SE, which may result in vasogenic edema formation via neurovascular interactions.

Seizure activity rapidly increases the synthesis and release of TNF-α, which acts on endothelial cells and changes the BBB permeability [11]. TNF-α is expressed at low levels in the normal brain and is rapidly up-regulated in glia, neurons and endothelial cells in various pathophysiological conditions [37]. TNF-α exerts various effects on brain function depending on its local tissue concentration, target cell type, and the specific receptor subtype. These subtypes include TNF receptor I or p55 receptor (TNFp55R), and TNF receptor II or p75 receptor (TNFp75R) [4,11,38,39]. Recently, we reported that an impairment of endothelial cell function via TNF-α mediated p65-Thr 485 NFκB phosphorylation is involved in SE-induced vasogenic edema, which result in extensive neutrophil infiltration and neuron-astroglial loss via TNFp75R [11]. In the present study, the basal level of TNF-α concentration was 106.1 pg/ml in the PC, although TNF-α expression was undetectable in normal brains. This TNF-α induction may be due to surgical injury during the insertion of the guide cannula for the microdialysis probe. However, SE significantly induced TNF-α synthesis and released it into brain parenchyma, and the TNF-α concentration was 151.8 pg/ml. Furthermore, sTNFp55R and SN50 pretreatment attenuated SE-induced vasogenic edema via the preservation of SMI-71. These findings indicate that the TNF-α/NFκB-mediated neuroinflammatory responses in the brain parenchyma play a crucial role in BBB disruptions following SE. However, sTNFp55R pretreatment did not completely reduce the volume of SE-induced vasogenic edema. Although TNF-α has been shown to directly increases BBB permeability in various experimental disease models [40,41], these findings indicate that TNF-α itself may not be the only upstream modifier of vasogenic edema development.

ET-1 is one of the potent and long-lasting vasoconstrictors that work in a paracrine and autocrine fashion. ET-1 binds to the ET_A_ receptor that expresses in smooth muscle cells within the cerebral vasculature [42]. Therefore, several lines of evidence indicate that ET-1 is an important mediator of cortical brain damage in terms of its potent vasoconstriction action inducing the decline of cerebral blood flow [43]. Thus, targeting ET-1 biosynthesis may be a strategy for preventing neurovascular injury. Although ET-1 is expressed in brain parenchyma, ET-1 cannot cross or alter the permeability of the BBB [44]. Therefore, it is likely that the neurovascular action of ET-1 as a vasoconstrictor would be limited during vasogenic edema formation. However, ET-1 also acts as vasodilator when it binds to the ET_B_ receptor. The ET_B_ receptor is predominantly expressed in neurons, glial cells, and capillary endothelial cells [22,23]. ET_B_ receptor activation in endothelial cells results in vasodilation via NO production, which causes rapid and short-lived vasodilation [45,46]. In the present study, we found up-regulated ET-1 expression in endothelial cells prior to SE-induced vasogenic edema formation via the TNF-α/NFκB-mediated pathway. Furthermore, ET-1 stimulated eNOS to synthesize NO in endothelial cells through the ETB receptor. In turn, NO-dependent reactive nitrogen species-mediated reduction in SMI-71 expression resulted in BBB disruption, leading to vasogenic edema. Therefore, our findings indicate that TNF-α-induced ET-1 expression in endothelial cells may be a potential factor to increase BBB permeability via ET_B_ receptor-mediated eNOS activation following SE.

In the present study, reductions in dystrophin/AQP4 expression correlated with the up-regulation of ET_B_ receptor in astrocytes after SE. These reductions were accompanied by an increase in 4-HNE (not NT) levels in astrocytes. Interestingly, Dong et al. [47] reported that the blockade of ET_B_ receptors in murine cardiomyocytes significantly attenuated NADPH oxidase subunit expression (p47phox and p67phox) and intracellular superoxide generation. The present study also showed that SE increased p47phox expression in astrocytes accompanied by increased 4-HNE levels. Furthermore, BQ788 and apocynin pretreatment effectively attenuated reductions in dystrophin/AQP4 expression in astrocytes by SE. Therefore, these findings reveal that ET_B_ receptor activation may increase intracellular ROS levels by NADPH oxidase in astrocytes, thereby inducing astroglial dysfunction that may affect vasogenic edema severity [4].

**Figure 7:**
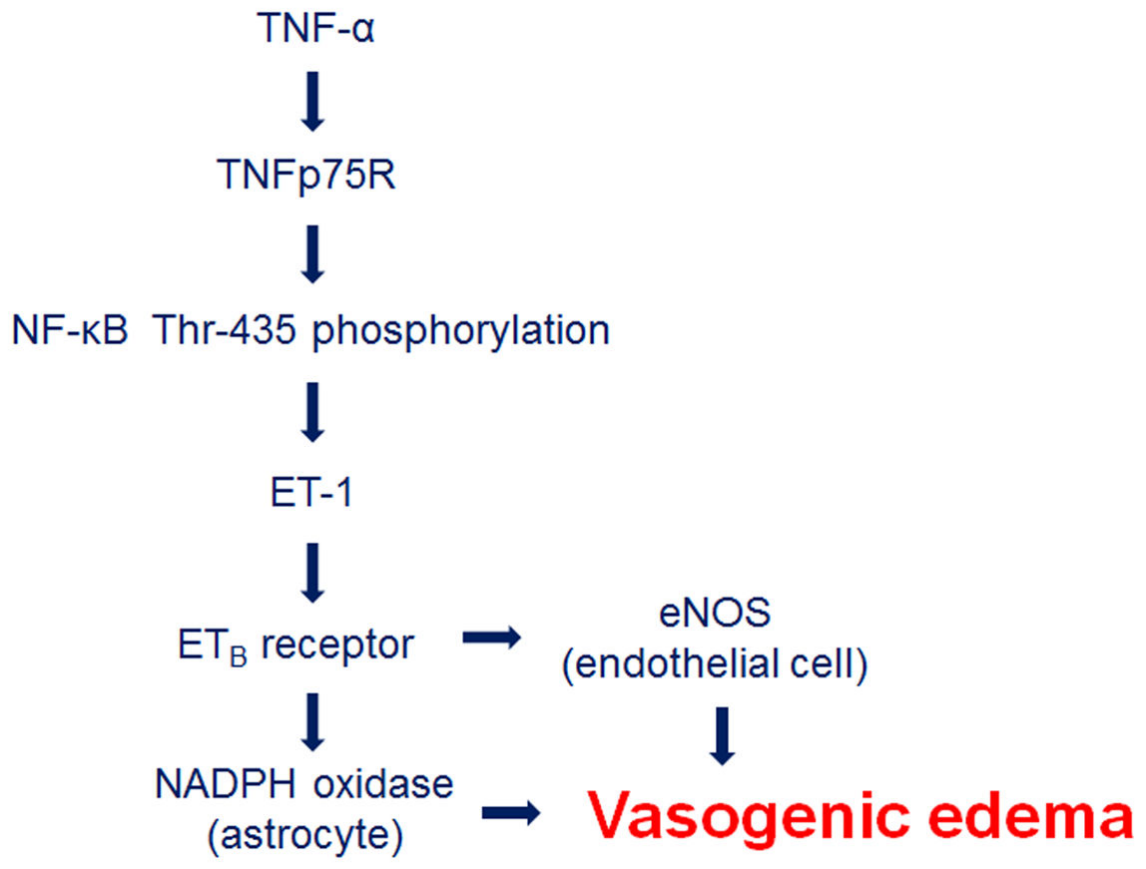
Scheme depicting the role of the ET-1 in vasogenic edema formation induced by SE.

In conclusion, our findings reveal that SE may result in the impairment of BBB function by endothelial-astroglial interactions through the TNF-α-ET-1-eNOS/NADPH oxidase pathway. Therefore, we suggest that the modulation of these ET_B_ receptor-mediated interactions may be an effective therapeutic strategy for vasogenic edema in various neurological diseases (Figure 7).
